# The succession of One Day Surgery with Breast Cancer Home Recovery Program by the National Cancer Institute (ODS BHR NCI) during the COVID-19 pandemic in Thailand

**DOI:** 10.1016/j.sopen.2024.12.001

**Published:** 2024-12-15

**Authors:** Chayanoot Rattadilok, Napaphat Poprom, Vipavee Niyomnaitham, Oradee Phadhana-anake, Jirawadee Ruamjaroenchai, Napat Saigosoom, Wimonporn Papathe, Suttida Thuranutch, Araya Chanwet

**Affiliations:** a Division of Breast Cancer Surgery, Department of Surgery, National Cancer Institute, Bangkok, Thailand; b Faculty of Public Health, Chiang Mai University, Chiang Mai, Thailand

**Keywords:** Breast cancer, One day surgery, Multidisciplinary team, Success, Surgical procedure

## Abstract

**Background:**

Breast cancer is the most common cancer among women. In response to the need to hospital stays and minimize waiting time for surgery, particularly during the COVID-19 pandemic, the National Cancer Institute developed the One Day Surgery with Breast cancer Home Recovery program (ODS BHR NCI). The aim of study is to assess the success rate of breast cancer surgeries conducted through this program and to evaluate the incidence of complications.

**Method:**

The cohort study includes all breast cancer patients who underwent surgery through the ODS BHR NCI program between August 2021 to November 2023. Eligible participants were under 70 years of age. The patients received comprehensive care and education from a multidisciplinary team, adhering to the program's guidelines. Their postoperative outcomes were monitored on the 1^st^, 3^rd^, and 5^th^ days following discharge.

**Result:**

A total of 206 patients participated in the ODS BHR NCI program during the COVID-19 pandemic in Thailand. The mean age and BMI were 54.21 ± 9.74 years and 24.18 ± 4.02, respectively. The success rate was 99.02 %, with only one case involving a serious complication. Minor complications were reported in 20 cases, including anesthesia-related issues and minor surgical complications. Notably, no patients were diagnosed with COVID-19 following their surgery.

**Conclusion:**

Breast cancer surgeries performed under the ODS BHR NCI program achieved a high success rate. The development of pre-, intra-, and post-operative care protocols, alongside comprehensive patient education, has potential to reduce complications and provide an effective model for extending systemic care to a wider range of patients.

## Highlights

• One Day Surgery (ODS) in breast cancer patients who underwent same-day surgery
• Breast cancer Home Recovery program (BHR): pre-, during-, and post-surgery care and providing guidance to breast cancer patients reduce surgical complications.
• Cancer anywhere, which allows cancer patients to receive treatment at any hospital in Thailand.

## Introduction

Breast cancer is currently the most commonly diagnosed cancer among Thai women, with the number of new cases rising dramatically each year. To address this issue, Thailand has implemented the “Cancer Anywhere” program, which enables cancer patients to receive treatment at any hospital across the country. As a result, the National Cancer Institute (NCI) has experienced an increase in the number of patients seeking treatment. Historically, the National Cancer Institute performed an average of 200–250 breast cancer surgeries annually.

Significant advancements have been made in both the diagnosing and treating breast cancer. Current treatment options include surgery, chemotherapy, targeted therapy, hormone therapy, and radiation therapy, each aimed at improving survival rates, reducing local recurrence of cancer, and enhancing the quality of life for patients. Surgical treatment for breast cancer typically involves two main components: breast surgery and axilla lymph node surgery. The breast surgery options include total mastectomy, breast-conserving surgery (BCS), and mastectomy with breast reconstruction. The axilla lymph node surgery options include sentinel lymph node biopsy (SLNB) and axillary lymph node dissection (ALND). It is estimated that only 20–30 % of patients with early-stage breast cancer with lymph node metastasis [[1], [2], [3], [4]]. However, post-surgical complications such as seroma, hematoma, infection, wound dehiscence, pain, numbness, frozen shoulder, and lymphedema are possible, as are anesthesia-related complications, including nausea, vomiting, and sore throat due to intubation [[1], [2], [3],^5^,^6^]. Despite advancements in breast cancer treatment, the management of potential surgical complications remains a crucial aspect of patient care.

Traditionally, post-surgery hospital stays lasted 3–7 days to monitor for complications and prepare for subsequent treatment. A previous study examined 72 cases of outpatient total mastectomies, where patients typically stayed in the hospital for 4–6 hours post-surgery. Within 30 days, five patients returned to the emergency room—three due to pain, which was managed with painkillers, and two due to complications requiring re-admission, such as infections [7]. Another study analysed breast cancer patients who underwent same-day surgery with a home recovery program. Success factors for the program included surgeries performed by breast surgeons and the use of multimodal pain management. Factors that led to re-hospitalization included patients classified under the American Society of Anesthesiologists (ASA) Class 3 to 4 and African American patients. Proper patient selection was found to the success rate of the program [8].

In 2019, the COVID-19 pandemic began to spread globally, with Thailand's outbreak starting in January 2021. The widespread transmission led hospitals to allocate significant resources to COVID-19 patients, complicating surgical preparations and limiting both medical personnel and other resources. Consequently, many hospitals had to suspend surgeries, and even when surgeries were possible, operating rooms and patient wards could not function at full capacity. This situation caused patients to be transferred to hospitals that could provide surgical services, leading to delays in cancer surgical treatment. However, the number of cancer patients requiring surgery remained unchanged, waiting times for surgery increased. The NCI faced similar challenges, with a consistent number of healthcare providers and operating rooms but fewer available beds, as some resources were diverted to support COVID-19 care. These disruptions in breast cancer treatment risked delays in patient care, which could affect survival rates, recurrence rates, and overall quality of life.

**Fig. 1:**
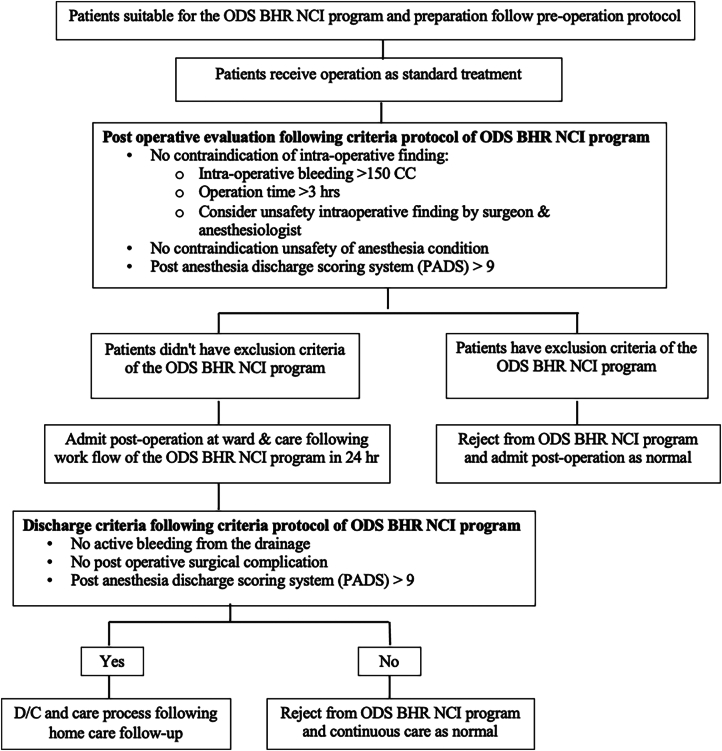
The flow of participants for the ODS BHR NCI program.

In response to the challenges posed by COVID-19, the NCI recognized the need to reduce hospital stays, shorten surgery wait times, and increase the efficiency of operating rooms. To address these issues, the NCI developed the “One Day Surgery with Breast cancer Home Recovery program by the National Cancer Institute (ODS BHR NCI)” to alleviate the backlog of breast cancer patients during the pandemic. The aim of this study was evaluated the success of the ODS BHR NCI program, assess the complication rates, and investigated the factors influencing the success rate and complications in breast cancer patients treated during the COVID-19 pandemic in Thailand (Fig. 1).

## Method

This cohort study was conducted on all breast cancer patients enrolled in the One Day Surgery with Breast cancer Home Recovery program by the National Cancer Institute (ODS BHR NCI) during the COVID-19 pandemic in Thailand, from August 2021 to November 2023. Upon being diagnosed with breast cancer, patients were provided with standard treatment and surgical planning. Each patient was assessed for eligibility to participate in the ODS BHR NCI program. The surgical options available to breast cancer patients included breast conserving surgery (BCS), mastectomy, sentinel lymph node biopsy (SLNB), and axillary lymph node dissection (ALND), with the following the patient's selection criteria (see Table 1). The study, along with all associated documents—including the consent form—was reviewed and approved by the ethics committee of the NCI (EC 65027, project number 027_2002RC_IN761). For each patient, guidance and documentation about the study's purpose and procedures were provided before obtaining informed consent. If a patient expressed interest in participating, they received counselling from a multidisciplinary team, including a surgeon, anesthesiologist, outpatient nurses, operating room nurses, and anesthesia nurses. A joint evaluation ensured patient safety, based on the study's inclusion and exclusion criteria (see Table 1), and each patient provided signed consent to participate.

**Table 1:** Show the inclusion and exclusion criteria for the ODS BHR NCI program.

Inclusion criteria
Age ≤ 70 year and ECOG 0–1
Absent morbid obesity (BMI < 35 kg/m^2^)
ASA class I-II or ASA class III but well control e.g., DM well controlled: BS < 250 mg/dl, HT well controlled: BP < 160/100 mmHg
No life-threatening comorbidities e.g., acute MI, COPD exacerbation, stroke
No history uses the narcotic drugs or psychotic drug e.g., MAOI
No history of anesthesic drug allergy
Availability of a relative or caregiver on the day of surgery and postoperatively
Patients able to perform self-care and accept to participate this program


Exclusion criteria for pre-operation	Exclusion criteria for intraoperation
Having history of malignant hyperthermia	Contraindication of intraoperation
Having respiratory tract infection	• Blood loss >150 CC
• Upper respiratory tract infection within 2 weeks	• Operation time > 3 hours
• Lower respiratory tract infection within 4 weeks	• Unsafety intraoperative finding by MDT team
Having difficult airway (ventilation/intubation)	Contraindication of anesthesia condition
Having OSA or use the CPAP	Post anesthesia discharge scoring system (PADS) < 9
Pain control after operation by oral tablets	**Exclusion criteria for post-operation**
Pregnancy	Active bleeding from the drainage
Multiple co-morbidity	Post operative surgical complication
Absent blood transfusion	Post anesthesia discharge scoring system (PADS) < 9

### Intervention and outcomes

#### Pre-operation phase

Before surgery, patients are assessed by a team of surgeons and anesthesiologists to determine their eligibility for the ODS BHR NCI program. If the evaluation indicates that participation is safe and appropriate, and the patient consents, the following steps were including:

*Knowledge assessment* was defined as patients understanding of the program and procedures was initially evaluated through a pre-test assessment.

*Patient education* was defined as comprehensive information and practical guidelines were provided on self-care before, during, and after surgery. This includes a home care program provided by the multidisciplinary team comprising surgeons, nurses, physical therapists and anesthesiologists. The team educated patients on the surgical preparation process, anesthesia options, potential benefits, risks, and possible complications.

*Post-education assessment* was defined as after receiving education, patients' understanding was reassessed to ensure preparedness for the surgery and home recovery process.

*Appointment scheduling* was defined as surgery and COVID-19 testing dates were scheduled following the National Cancer Institute's protocols.

#### On the day of surgery

On the day of surgery, patients fasted from midnight and arrived at the hospital as scheduled. A COVID-19 test was performed on the day of surgery for patients undergoing surgery between August 2021 to June 2023, per the Ministry of Public Health's guidelines. After the COVID-19 test, patients are prepared for surgery by the anesthesiologist and then taken to the operating room.

*Anesthesia administration* was defined as either general or local anesthesia was administered depending on the patient's surgical plan.

*Surgical procedure* was defined as surgery proceeds according to the treatment plan, consisting of: **breast surgery** options included mastectomy or BCS. In BCS cases, patients typically did not require drainage tubes, while mastectomy patients had drainage tubes placed. **Axillary lymph node surgery** options included SLNB or ALND. In SLNB, dye or radioactive isotope was injected to identify the sentinel lymph node (SLN) in the axilla. If no cancer cells were detected in the SLN, further lymph node dissection was not performed. If cancer cells were detected, level 1 and 2 axillary lymph nodes were dissected (ALND), and a drainage tube was placed.

*Evaluation exclusion criteria during surgery* was defined as after surgery; the surgical wounds were closed with absorbable sutures per standard procedures. Patients exhibiting certain intraoperative conditions were excluded from the ODS BHR NCI program to ensure patient safety and treatment efficacy.

#### Post-operation phase

Patients were monitored in the recovery room for 1–2 hours before being transferred to the patient ward for up to 24 hours.

*Multidisciplinary team assessment* was defined as the multidisciplinary team assessed the patient's condition post-surgery. If the patient was stable, discharge preparations began.

*Discharge planning* was defined as detailed self-care instructions were provided, covering wound care, drainage management, pain management, and emergency contact procedures for post-surgical support.

#### Home care and follow-up

This comprehensive follow-up approach ensures that patients receive consistent care and support, promoting a smooth and safe recovery process at home. The multidisciplinary team ensures continuous patient care after discharge through a structured follow-up plan:

*Initial follow-ups* were defined as the multidisciplinary team monitored patients via telemedicine on days 1^st^, 3^rd^, and 5^th^ post-surgeries. The team checked vital signs, surgical wound condition, drainage tube management, and shoulder mobility. Patients were given dietary advice, wound care instructions, and guidance on managing the drainage tube and pain.

*Post-surgery follow-up appointment* was defined as patients were scheduled for a follow-up visit approximately 7–10 days after surgery and continued follow-ups occurred every 1–2 weeks for one month. A questionnaire was used to assess patient satisfaction, which included rating scales and open-ended questions. The questionnaire assessed patients' overall satisfaction with the services and care provided by the ODS BHR NCI program. In addition, it incorporated detailed evaluations of specific aspects and components of the multidisciplinary team. These evaluations examined satisfaction with the services and care provided by surgeons and anesthesiologists, as well as those provided by other multidisciplinary team members. These team members included outpatient nurses, inpatient nurses, nurse anesthetists, operating room nurses, physiotherapists, and pharmacists.

*Standard care monitoring* was defined as the multidisciplinary team follows standard protocols to ensure that any issues were addressed promptly, maintaining patient safety and satisfaction throughout the recovery period.

### Data collection & analysis

Data collection encompassed several aspects of patient care, including basic patient information (demographics, medical history, and other relevant details), surgical information (types of surgery, operation time, and length of hospital stay), pathological result, and follow-up information (post-operative care, complications, and patient satisfaction). All data analysis were analysed using STATA version 14.

For categorical data, such as the success rates of the ODS BHR NCI program and post operative complication, the Chi-square test or Fisher's exact test was used, and the results were reported as the number with percentages. Continuous variables, including operation time and length of hospital stay, were analysed using the student *t*-test or Mann-Whitney test, with the results reported as means with standard deviations or medians with interquartile ranges, depending on the distribution. The logistic regression was performed to explore the strength and significance of the relationships between success rates/complications and various factors. A *p*-value of <0.05 was considered indicative of a statistically significant difference or relationship.

## Result

According to the study of all breast cancer patients who participated in the ODS BHR NCI program during the COVID-19 pandemic in Thailand (August 2021 – November 2023), a total of 206 female patients were included, with an average age was 54.21 ± 9.74 years. Among them, 94 patients (45.63 %) had underlying diseases, mostly hypertension, diabetes, and hyperlipidemia. Most patients were classified as ASA 1–2 groups (ASA 1; 55 % and ASA 2; 45 %) and there average BMI was 24.18 ± 4.02. The patients came from various regions of Thailand, with the majority from Bangkok and its surrounding areas (71.84 %), followed by the central and southern regions. Additionally, 7 foreign patients from Cambodia and Myanmar were included. The majority (97.09 %) of patients had breast cancer, mostly in stages 1–2 (71.5 %), with an average tumor size was 1.9 cm (range 0, 8.5 cm). Most tumors were invasive ductal carcinoma, with 78.15 % classified as T1–2. The average total number of lymph nodes removed was 5 (range 1, 41), with an average of 0 lymph nodes showing metastasis (range 0–18). Three patients (1.45 %) had phyllodes tumors, and three others (1.45 %) were *BRCA 1/2* mutation carrier who underwent prophylactic mastectomy.

Mastectomy was performed in 52.55 % of cases, followed by BCS in 44.9 %. Other surgical interventions, such as implant reconstruction and mastopexy, were also performed. Among axillary lymph node surgeries, SLNB) was performed in 66.67 % of cases, while axillary lymph node dissection (ALND) was performed in 33.33 %. A combination of SLNB and ALND was performed in 40 % of ALND cases, while 60 % underwent ALND alone. Neoadjuvant treatment was given to 43 patients (20.87 %), primarily in the form of neoadjuvant chemotherapy (97.67 %) using anthracycline-based and taxane-based regimens. Additionally, 9.3 % of patients received neoadjuvant chemotherapy with target therapy (see Table 2).

**Table 2:** Demographic data of patients.

Characteristic	*N* = 206
Age (years), mean (SD)	54.21 ± 9.74
Having underlying, n (%)	94 (45.63 %)
BMI, mean (SD)	24.18 ± 4.02
Breast cancer staging (*n* = 200)	
• Stage 0	15 (7.5 %)
• Stage 1	71 (35.5 %)
• Stage 2	72 (36 %)
• Stage 3	31 (15.5 %)
• Stage 4	7 (3.5 %)
• NA	4 (2 %)
Tumor size (cm), median (range)	1.90 (0.00, 8.50)
Number of LN metastasis, median (range)	0.00 (0.00, 18.00)
Total number LN, median (range)	5.00 (1.00, 41.00)
Experience of breast surgeon	
• Experience of breast surgeon <5 years	37 (17.96 %)
• Experience of breast surgeon ≥5 years	169 (82.04 %)
Breast operation (*n* = 196)	
• Mastectomy	103 (52.55 %)
• BCS	88 (44.90 %)
• Other	5 (2.55 %)
Axilla operation (*n* = 180)	
• SLNB	120 (66.67 %)
• ALND	60 (33.33 %)
Having neoadjuvant treatment (n = 206) • Neoadjuvant chemotherapy (*n* = 43)	43 (20.87 %) 42 (97.67 %)
• Neoadjuvant target therapy (n = 43)	4 (9.3 %)
• Neoadjuvant hormonal therapy (n = 43)	2 (0.46 %)
Operation time (min), mean (SD)	72.61 ± 22.93
Blood loss (ml), median (range)	10.00 (1.00, 100.00)
Type of patients' ward	
• General ward	162 (78.64 %)
• Private ward	44 (21.36 %)

Most surgeries (99.03 %) were conducted under general anesthesia, with only 2 patients receiving total intravenous anesthesia (TIVA) and local anesthesia, one of whom had scoliosis and was at high risk for general anesthesia. Five surgeons were involved in the operations, three of whom had over five years of experience, while the remaining two had less. The average operation time was 72.61 ± 22.93 min, with an estimated blood loss was 10 ml (range 10, 100 ml). Postoperative pain scores in the recovery room and patient ward were 2 (range 0, 5) and 2 (range 0, 4), respectively. Among BCS patients, 47 (22.82 %) received postoperative local anesthesia. An average of 131 patients (63.59 %) were discharged with drainage tubes, with 72.52 % having one tube and 27.48 % having two tubes, which remained for about 1–2 weeks (see Table 3).

**Table 3:** Show the success rate and the complication of the ODS BHR NCI.

Characteristic	n (%)
Success rate of the ODS BHR NCI program	204 (99.02 %)
Complication	21 (10.19 %)
• Serious complication	1 (0.49 %)
• Non-serious complication	20 (9.70 %)
o Surgical complication	17 (8.25 %)
o Anesthetic complication	3 (1.46 %)
Non-serious surgical complication	
• Pain score > 4 (*n* = 17)	12 (70.59 %)
• Limit ROM 120–150 (n = 17)	5 (29.41 %)
• Lymphedema	0 (0 %)
• Infection	0 (0 %)
• Wound dehiscence	0 (0 %)
Re-operation	1 (0.49 %)
Re-admission	0 (0 %)
Test score, mean (SD)	
• Pre-test score	7.03 ± 1.27
• Post test score	9.10 ± 0.83
The overall patient satisfaction of the ODS BHR NCI program, mean (SD)	8.44 ± 0.72

The success rate for breast cancer surgery under the ODS BHR NCI program was 99.02 % (204 cases). Most patients (96.6 %) had a hospital length of stay (LOS) within 24 hours, and 5 patients (2.43 %) had stays of 8 hours. Two patients failed to participate fully in the ODS BHR NCI program: one due to the need for re-operation and the other because of age and living conditions. The latter patient was elderly and lived in a rural area. This patient, one of the first in the study, was not proficient with electronic communication devices used to contact the MDT team. Moreover, the patient's caregiver could only assist in the evening, leaving the patient anxious about managing the drainage tube during the day, which led to refusal of discharge within 24 hours. The majority of patients stayed in the general ward (78.64 %). Pre-test and post-test scores averaged 7.03 ± 1.27 and 9.1 ± 0.83, respectively, indicating increased self-care knowledge. Moreover, the patients' satisfaction assessed by the questionnaire. It assessed overall satisfaction with the services and care provided under the ODS BHR NCI program and included specific evaluations of individual aspects and components of the multidisciplinary team. The overall patient satisfaction averaged 8.44 ± 0.72.

There were 21 cases of complications (10.19 %), with only 1 serious complication (0.49 %), requiring re-operation due to hematoma within 24 hours. The remaining 20 cases were minor surgical or anesthesia-related complications that resolved within 72–120 hours. No cases of surgical site infection, lymphedema, or COVID-19 infection were reported during or after surgery. All patients managed their drainage tubes without displacement or re-admission. Among the 20 minor surgical complications, 3 were anesthesia-related complications (1.46 %) and 17 were minor surgical complications (8.25 %). Anesthesia-related complications included nausea, vomiting, and postoperative sore throat. Among the 17 minor surgical complications, 12 involved postoperative pain (pain score > 4), and 5 involved limited shoulder range of motion (120–150 degrees), which resolved within 5–7 days post-surgery (see Table 3).

Factors associated with surgical failure in the ODS BHR NCI program included patient age, pre-test and post-test scores, type of surgery, surgeon's experience, BMI, underlying diseases, postoperative pain, type of patients' ward, and complications. However, with only 2 cases of surgical failure, these factors should be interpreted with caution (see Table 4).

**Table 4:** Show the factors affecting the success rate of the ODS BHR NCI.

Variable		Failure (n = 2)	Success (*n* = 204)	*p*-value
Age (years), mean (SD)		54.08 ± 9.69	68.00 ± 4.24	0.044
Age group (years), n (%)	< 40	0 (0.00)	20 (9.8)	0.024
	40–64	0 (0.00)	160 (78.43)	
	>64	2 (100.00)	24 (11.76)	
Underlying, n (%)	No underlying	1 (50.00)	118 (57.84)	1.000
	Having underlying	1 (50.00)	86 (42.16)	
Experience of surgeon, n (%)	< 5 years	1 (50.00)	167 (81.86)	0.336
	≥ 5 years	1 (50.00)	37 (18.14)	
Breast operation, n (%)	No	0 (0.00)	10 (4.90)	1.000
	Mastectomy	1 (50.00)	102 (50.00)	
	BCS	1 (50.00)	87 (42.65)	
	Other	0 (0.00)	5 (2.45)	
Axilla operation, n (%)	No	0 (0.00)	26 (12.75)	1.000
	SLNB	2 (100.00)	118 (57.84)	
	SLNB +ALND	0 (0.00)	24 (11.76)	
	ALND	0 (0.00)	36 (17.65)	
Complication, n (%)	Having complication	1 (50.00)	20 (9.80)	0.194
	No complication	1 (50.00)	184 (90.20)	
Pain score, n (%)	≤ 4 score	2 (100.00)	199 (97.55)	1.000
	> 4 score	0 (0.00)	5 (2.45)	
Post test score, n (%)	≤ 6 score	0 (0.00)	0 (0.00)	0.398
	7–8 score	1 (50.00)	45 (22.06)	
	9–10 score	1 (50.00)	159 (77.94)	

Additionally, among the 21 cases of complications, there was a preliminary association between certain factors, specifically post-test scores and inpatient ward type with postoperative complications, which was statistically significant (see Table 5). However, the risk factors associated with postoperative complications, type of patients' ward significantly influenced overall complications. Patients in the private wards had a 3.21 times higher risk (95 % CI; 1.25, 8.22) of complications compared to those in general wards. Other factors, such as surgery type, operation time, blood loss, surgeon's experience, age, BMI, underlying diseases, cancer stage, tumor size, number of lymph nodes with metastasis, number of lymph nodes removed, pre-test and post-test scores, postoperative pain, and number of drainage tubes, were not significantly associated with postoperative complications (see Table 6).

**Table 5:** Show the factors affecting the complication of the ODS BHR NCI.

Variable		Complication (*n* = 21)	No complication (*n* = 185)	*p*-value
Age (years), mean (SD)		56.61 ± 9.79	53.94 ± 9.73	0.234
Age group (years), n (%)	< 40	3 (14.29)	13 (7.03)	0.064
	40–64	13 (61.90)	151 (81.62)	
	>64	5 (23.81)	21 (11.35)	
BMI (kg/m2), n (%)	< 18.50	1 (4.76)	9 (4.86)	0.717
	18.50–25.00	10 (47.62)	103 (55.68)	
	25.00–30.00	9 (42.86)	56 (30.27)	
	≥ 30.00	1 (4.76)	17 (9.19)	
Underlying, n (%)	No underlying	11 (52.38)	108 (58.38)	0.598
	Having underlying	10 (47.62)	77 (41.62)	
Experience of surgeon, n (%)	< 5 years	18 (85.71)	150 (81.08)	0.771
	≥ 5 years	3 (14.29)	35 (18.92)	
Breast operation, n (%)	No	1 (4.76)	9 (4.86)	0.703
	Mastectomy	10 (47.62)	93 (50.27)	
	BCS	9 (42.86)	79 (42.70)	
	Other	1 (4.76)	4 (2.16)	
Axilla operation, n (%)	No	4 (19.05)	22 (11.89)	0.758
	SLNB	11 (52.38)	109 (58.92)	
	SLNB + ALND	2 (9.52)	22 (11.89)	
	ALND	4 (19.05)	32 (17.30)	
Pain score, n (%)	≤ 4 score	20 (95.24)	181 (97.84)	0.419
	> 4 score	1 (4.76)	4 (2.16)	
Post test score, n (%)	≤ 6 score	0 (0.00)	0 (0.00)	0.041
	7–8 score	1 (4.76)	45 (24.32)	
	9–10 score	20 (95.24)	140 (75.68)	
Operation time, n (%)	≤ 60 min	10 (47.62)	64 (34.59)	0.493
	60–110 min	10 (47.62)	108 (58.38)	
	> 110 min	1 (4.76)	13 (7.03)	
Type of patients' ward, n (%)	General ward	12 (57.14)	150 (81.08)	0.021
	Private ward	9 (42.86)	35 (18.92)	
Staging, n (%)	Stage 0–1	6 (30.00)	80 (44.69)	0.208
	Stage 2–4	14 (70.00)	99 (55.31)	
Neoadjuvant chemotherapy	Having NAC	3 (14.29)	39 (21.08)	0.464
(NAC), n (%)	No NAC	18 (85.71)	146 (78.92)	
Number LN dissection, n (%)	< 3	1 (5.56)	24 (14.29)	0.584
	3–10	11 (61.11)	95 (56.55)	
	≥ 10	6 (33.33)	49 (29.17)	
Retained drain, n (%)	No drain	8 (38.10)	67 (36.22)	0.949
	1 drain	9 (42.86)	86 (46.49)	
	2 drains	4 (19.05)	32 (17.30)	
ASA, n (%)	Class I	11 (52.38)	108 (58.38)	0.598
	Class II	10 (47.62)	77 (41.62)

**Table 6:** Show the factors affecting the complication of the ODS BHR NCI.

Variable		Odds ratio (95%CI)	*p*-value
Age, years		1.03 (0.98, 1.08)	0.236
Age group (years)	< 40	1	
	40–64	0.37 (0.09, 1.47)	0.161
	≥ 65	1.03 (0.21, 5.05)	0.969
BMI	< 18.50	1	
	18.5–24.9	0.87 (0.10, 7.61)	0.903
	25–29.9	1.44 (0.16, 12.82)	0.740
	≥ 30	0.52 (0.02, 9.49)	0.666
Underlying	No underlying	1	
	Underlying	1.27 (0.51, 3.15)	0.599
Experience of surgeon	< 5 years	1	
	≥ 5 years	1.4 (0.39, 5.01)	0.605
Type of patients' ward	General ward	1	
	Private ward	3.21 (1.25, 8.22)	0.015*
Pain score	≤ 4 score	1	
	> 4 score	2.26 (0.24, 21.24)	0.475
Post test score	≤ 6 score	–	
	7–8 score	1	
	9–10 score	6.42 (0.83, 49.25)	0.073
Staging	Stage 0–1	1	
	Stage 2–4	1.88 (0.69, 5.12)	0.214
Neoadjuvant chemotherapy	Having NAC	1	
(NAC)	No NAC	1.60 (0.44, 5.72)	0.467

## Discussion

Currently, the average number of breast cancer patients treated surgically at the NCI ranges from 200 to 250 cases annually, with post-surgical hospital stays average 3 to 7 days for monitoring complications and preparing for discharge. Our study, involving 206 breast cancer patients who participated in the ODS BHR NCI program during the COVID-19 pandemic (August 2021 to November 2023), demonstrated promising outcomes. Importantly, no patients required readmission or contracted COVID-19 during or after surgery.

The ODS BHR program showed a success rate of 99.02 %, a figure higher than previously reported success rates (63.78 % to 91.67 %) from similar studies [7,8]. Furthermore, our results align with those of Marla and Stallard's systematic review, which reported a success rate of 86–100 % across 11 studies of outpatient same-day breast cancer surgeries [9]. In our study, 96.6 % of patients had a length of stay (LOS) of less than 24 hours, with 2.43 % of patients discharged after only 8 hours—comparable to findings by Mohamed-Aly Bakeer et al., where patients had an average LOS of 4 to 6 hours [7,8].

Common postoperative complications in breast cancer surgeries include infection, lymphedema, hematoma, numbness, reduced shoulder range of motion, and frozen shoulder [10,11]. Our study reported a relatively low overall surgical complication rate of 10.19 %, which is lower than previous studies [12,13]. The incidence of hematoma in this study was only 0.49 %, lower than those found in previous studies [[12], [13], [14]]. Similarly, a study by Posteki G., conducted during the COVID-19 pandemic, found that early-discharge patients had fewer complications, including a hematoma rate of 3.7 % [15].

Our study also reported no cases of infection or lymphedema, in contrast to previous studies, where infection rates ranged from 3.16 to 15 % and lymphedema rates of 9.4 % [[12], [13], [14]]. Furthermore, no patients in our study experienced issues with requiring readmission or drainage tube displacement, whereas previous research cited a 1.2 % readmission rate due to drainage issues [16]. Among non-serious complications, 20 patients experienced issues such as pain and limited range of motion, which resolved within 72–120 hours post-surgery.

Regarding factors influencing surgical success in previous studies include age, BMI, race, ASA classification, pain level, multi-modal pain management, type of surgery, operation time, and surgeon's experience [7,8]. In our study, only 2 patients did not fully participate in the ODS BHR NCI program. When considering factors associated with the failure of this program (e.g., age, race, pre-test and post-test scores, type of surgery, surgeon's experience, BMI, comorbidities, postoperative pain score, and complications), limiting the identification of definitive factors related to surgical failure. A larger sample size may yield more significant insights into variables impacting success rates. Brooke Vuong, et al. identified factors such as multi-modal pain management and surgeries performed by breast surgery specialists (> 50 breast surgeries/year) as contributing to success. Specifically, surgeries performed by experts increased success rate by approximately 2.12 (95 % CI; 1.42, 3.18) [8]. Bilateral mastectomy reduced the success rate 0.7 times (95 % CI 0.54, 0.91), and ASA class 3–4 by 0.69 times (95 % CI 0.54, 0.87).

Regarding factors influencing postoperative complications, our study identified a preliminary association between postoperative complications and factors such as post-test scores and the type of patients' ward. The type of ward significantly impacted overall complications. However, our study included patients with comorbidities, particularly diabetes, as well as older and obese patients. Other potential risk factors, such as surgery type, operation time, blood loss, surgeon experience, age, BMI, comorbidities, cancer stage, tumor size, number of metastatic lymph nodes, preoperative treatment, pre-test and post-test scores, postoperative pain, and the number of drainages tubes, did not significantly impact complication rates. These findings differ from the previous studies, which reported that age, ASA classification, diabetes, BMI, smoking, obesity, wound classification, extensive tissue dissection, insertion of drainage tubes, seroma, longer operation time and inpatient vs. outpatient surgery were relevant risk factors [13,14,17].

In terms of surgical procedures, our study found no significant differences in complication rates between mastectomy and BCS patients. This contrasts with previous studies that showed mastectomy and ALND patients had higher rates of surgical site complications [13,14]. The most common complication associated with axilla operation was reduced shoulder range of motion. The previous study found that patients who underwent BCS or mastectomy with SLNB or ALND, and who received physical therapy starting on the first day, in addition to preoperative education one week before surgery, showed no significant differences in shoulder flexion and abduction at 2 and 6 weeks post-surgery compared to preoperative measurements in SLNB patients [18]. However, ALND patients showed reduced shoulder flexion and abduction at 2 weeks but improved abduction at 6 weeks compared to preoperative measurements (*p* = 0.0016), with no significant difference in flexion at 6 weeks. In our study, all patients received preoperative education on self-care, including shoulder rehabilitation exercises. As a result, only 5 cases experienced limited ROM (120–150 degrees), and all improved within 72–120 hours post-surgery, with no cases of frozen shoulder.

Regarding complications, our study identified preliminary relationships between post-test scores, patient ward type, and postoperative complications. Patients in private ward had a 3.21 times higher risk of complications compared to those in the general ward. This may be due to differences in the care environment: general wards are often better equipped for surgical patients, providing specialized postoperative care that might reduce complication rates. General ward patients benefit from group-based education and peer learning, which may improve their confidence in self-care practices. For example, post-surgical shoulder exercises might be better adhered to in a general ward setting, peer support fosters more consistent engagement with rehabilitation.

This group-based learning dynamic contrasts with private wards, where individualized care is provided, but perhaps without the same level of peer encouragement. Therefore, the findings suggest that combining individual and group-based care in patient management may reduce complications and foster better outcomes. This is in line with findings from Posteki G., where preoperative education, self-care instructions, and multidisciplinary team communication were shown to significantly reduced postoperative complications, treatment costs, and medical staff workload [15].

Despite these promising results, the small sample size may limit the generalizability of our findings. Expanding the number of participants could further elucidate factors influencing complications and success rates. Additionally, future studies could explore the adaptation of this program for other conditions, using the ODS BHR model as a template for managing patients with different diseases in a post-pandemic healthcare environment.

## Conclusion

The One Day Surgery with Breast cancer Home Recovery program by the National Cancer Institute, implemented to reduce overcrowding during the COVID-19 pandemic, achieved a success rate of 99.02 %. The average patient satisfaction score was 8.44 ± 0.72, with no cases of COVID-19 infection during or after surgery. This approach demonstrates the potential to reduce surgical complications and could be adapted into a systemic innovation for managing patients with other diseases.

## Funding source statement

This research did not receive any specific grant from funding agencies in the public, commercial, or not-for-profit sectors.

## CRediT authorship contribution statement

**Chayanoot Rattadilok:** Writing – review & editing, Writing – original draft, Visualization, Validation, Supervision, Resources, Project administration, Methodology, Investigation, Funding acquisition, Formal analysis, Data curation, Conceptualization. **Napaphat Poprom:** Writing – review & editing, Validation, Software, Conceptualization. **Vipavee Niyomnaitham:** Writing – review & editing, Resources, Conceptualization. **Oradee Phadhana-anake:** Writing – review & editing, Resources, Conceptualization. **Jirawadee Ruamjaroenchai:** Writing – review & editing, Resources, Conceptualization. **Napat Saigosoom:** Writing – review & editing, Resources, Conceptualization. **Wimonporn Papathe:** Writing – review & editing, Resources, Conceptualization. **Suttida Thuranutch:** Writing – review & editing, Resources, Conceptualization. **Araya Chanwet:** Writing – review & editing, Resources, Conceptualization.

## Ethical approval statement

Certificate of approval by Research Committee of National Cancer Institute: Project number 027_2022RC_IN761.

## Declaration of competing interest

The authors declare that they have no known competing financial interests or personal relationships that could have appeared to influence the work reported in this paper.
